# The comparison of capnography and epigastric auscultation to assess the accuracy of nasogastric tube placement in intensive care unit patients

**DOI:** 10.1186/s12876-020-01353-5

**Published:** 2020-06-22

**Authors:** Elahe Heidarzadi, Rostam Jalali, Behzad Hemmatpoor, Nader Salari

**Affiliations:** Department of Nursing, Faculty of Nursing and Midwifery, Kermanshah University of Medical Sciences, Kermanshah, Iran

**Keywords:** Nasogastric tube, Intensive care unit, Treatment outcome, Human, Adult

## Abstract

**Background:**

Placement of nasogastric (NG) tubes is a common procedure for patients especially in intensive care units (ICUs). Thus, it is important to determine the correct placement of the tube to prevent misplacement in the airways. Accordingly, the aim of this study was to compare the epigastric auscultation and capnography in assessing the accuracy of NG tube insertion in ICU patients.

**Methods:**

In this descriptive comparative study, 60 patients were selected trough convenience sampling. After insertion of the NG tube in a standard method, the accuracy of placement of the tube with both epigastric auscultation and capnography was investigated. The NG tube insertion accuracy was then confirmed via radiography. Data analysis was performed using statistical software SPSS version 23.

**Results:**

The result showed that capnography had a sensitivity, specificity, and accuracy of 100, 92.5, and 95% respectively, but epigastric auscultation had 90, 80, and 83.4% respectively. The Kappa agreement coefficient between two methods was − 0.759.

**Conclusion:**

The results revealed that the use of the capnography is preferable over the epigastric auscultation to confirm the correct insertion of the NG tube. It is recommended that more than one method be applied to detect and confirm the correct insertion of the NG tube.

## Background

Patients admitted to the emergency department and the intensive care units (ICUs) with a reduced level of consciousness in general often require nasogastric (NG) tube insertion [1]. The NG tubes are widely used in special sectors, with the annual application of this equipment being 1.2 million in the United States [2] and more than 1 million in the UK [3]. In general, NG tube insertion is carried out with various diagnostic and treatment purposes [4]. Typically, one of the most important goals of using these tubes is to supply nutritional needs [5]. Other applications of these tubes include drug therapy and reducing stomach pressure, especially in abdominal operations [6].

Despite all the benefits of NG tubes, their inappropriate placement could endanger the safety of patients [7]. Most insertions are unsafe and lead to the accidental entry of NG tubes into airways and routes other than the gastrointestinal tract [3, 8]. Airway misplacement of NG tubes is the most common issue in this regard [9]. With the incorrect placement of NGs, about 3600–8400 cases of lung injuries and 1200–3600 deaths occur in the United States on an annual basis [2]. Misplacement of NG tubes causes serious problems, including aspiration, pneumothorax, pneumonia, bronchopleural fistula, emphysema, pulmonary hemorrhage, mediastinal inflammation, esophageal perforation, atelectasis, and mortality [10].

Today, various clinical methods are employed to determine the location of the NG tube, some of which are not reliable [11] or not available at the patient’s bedside. These methods include measuring the bilirubin concentration, pepsin, and trypsin of aspirated materials [12]. A new method for locating NG tubes in patients is measuring the carbon dioxide output from the expiratory flow of the lungs, which is carried out by a device called capnograph [13]. Since carbon dioxide exists only in the expiratory air, capnography can be applied to distinguish the airway from the gastrointestinal tract in the tube placement= [14].

In a study, this method was introduced as a safe and cost-effective approach in evaluating the NG tube, which promotes patient care [15]. Meanwhile, another research considered this approach ineffective in this regard [16]. Some sources have described the use of a capnograph along with another method to ensure the correct insertion of the NG tube [15]. Another method for determining and confirming the location of the tube in patients is the epigastric auscultation, which involves instilling air into the feeding tube while using a stethoscope placed over the stomach. Some of the benefits of this technique include simplicity and cost-effectiveness [17, 18]. Nevertheless, different sources consider this traditional technique as an unreliable test [5]. While epigastric auscultation is most commonly applied by nurses [19] and is introduced as an unreliable traditional technique in some sources [20, 21], its superiority over capnograph has been shown in some studies [22, 23]. Thus, considering the failure to identify the desirable method in previous studies, this study aimed to compare capnography and epigastric auscultation in confirming the correct placement of the NG tube.

## Methods

This was a descriptive and comparative study performed in two intensive care units (ICUs) of hospitals of Kermanshah, Iran in June 2018 to June 2019 (The ICUs in the hospitals affiliated to Kermanshah University of Medical Sciences were evaluated). A total of 60 patients were selected through convenience sampling, and the sample size was estimated using the sample size formula along with the formula for estimating the ratio of a qualitative attribute with a 95% confidence level for all patients intubated.

To determine the sample size using the estimation formula of the ratio of a qualitative trait in the study population and with 95% confidence (α-1) and based on the study by Alpieren et al. [16]. Diagnosis of gastric tube insertion as well as the ratio of gastric nasal tube placement correctly diagnosed as gastric epigastric insertion were calculated as 0.95 below.

Sample Size Determination Based on Ayten Zaybak:

Gastric nasal tube placement ratio diagnosed correctly by gastric capnometry (sidestream) 0.95 and gastric nasal tube placement ratio diagnosed correctly by gastric epigastric method 0.90 is calculated as follows.

Research tools included demographic characteristics form (age, gender, disease diagnosis, and admitted hospital), physiological information checklist (blood pressure, pulse, and arterial oxygen saturation), and Glasgow Coma Scale, which is designed to evaluate the level of consciousness and responses of patients to stimuli which is carried out based on three behaviors of patients. The participants were enrolled in the study after selecting the target group and obtaining written consent from their legal guardians. The questionnaires were completed by the first author. Before the start of the experiment, the radiologist was informed to attend the patient’s bedside. The NG tube was inserted by the relevant nurse in a standard manner. The location of the tube was evaluated using the epigastric auscultation. In this regard, after the completion of the intubation, 10 ml of air was blown into the tube through a syringe attached to the end of the NG tube. Simultaneously, the air flow was listened to by the Littmann classic II S.E. Once the NG tube is impregnated with lidocaine gel, it is directed from one of the nasal passages to the esophagus, then to the stomach, and then to the NG fixes with the CM scalp.

Interventions for reducing the leak from the connection between NG tube and calorimetric capnography (sidestream) probe were done with connection. Standard CO2 levels were used for gastric ultrasound [24]. Stethoscope (made in the USA), and the presence or absence of the sound of the air motion was recorded.

$$ {\displaystyle \begin{array}{l}\alpha =0.05\to {Z}_{1-\raisebox{1ex}{$\alpha $}\!\left/ \!\raisebox{-1ex}{$2$}\right.}=1.96\\ {}P=0.95\\ {}d=0.1\\ {}n=\frac{{\left({z}_{1-\raisebox{1ex}{$\alpha $}\!\left/ \!\raisebox{-1ex}{$2$}\right.}\right)}^2P\left(1-P\right)}{(d)^2}=\frac{(1.96)^20.95\left(1-0.95\right)}{(0.1)^2}=\frac{(3.84)(0.0475)}{(0.1)^2}=\frac{0.1824}{0.01}=18.24\\ {}n\ge 18\\ {}\\ {}\alpha =0.05\to {Z}_{1-\raisebox{1ex}{$\alpha $}\!\left/ \!\raisebox{-1ex}{$2$}\right.}=1.96\\ {}P=0.90\\ {}d=0.1\\ {}n=\frac{{\left({z}_{1-\raisebox{1ex}{$\alpha $}\!\left/ \!\raisebox{-1ex}{$2$}\right.}\right)}^2P\left(1-P\right)}{(d)^2}=\frac{(1.96)^20.90\left(1-0.90\right)}{(0.1)^2}=\frac{(3.84)(0.09)}{(0.1)^2}=\frac{0.3456}{0.01}=34.56\\ {}n\ge 35\\ {}\\ {}\\ {}\end{array}} $$

Next, a capnograph adapter (Alborz B9 vital signs monitor, made in Iran) was connected to the end of the NG tube and the result was recorded after 1 min. In the final stage, chest radiography was performed to determine the exact location of the tube. The results of the other two techniques were confirmed or rejected according to the radiographic diagnosis. Data analysis was performed in SPSS version 23 using descriptive statistics (frequency distribution, mean and standard deviation of the variables) to estimate the sensitivity, specificity, and accuracy of capnography and epigastric auscultation techniques, and to compare the accuracy of the two methods.

## Results

The mean age of patients was 47.06 ± 19.11. Also, 83.3% of the patients were male and 95% of patients were admitted to the ICU; 35% had systolic blood pressure less than 120 and 48.3% had systolic blood pressure above 120 mmHg. Further, 45% had diastolic blood pressure less than 80 and 43.3% had diastolic blood pressure above 80 mmHg. Finally, 13.3% had a pulse rate below 60 and 63.3% had a pulse rate above 80 beats / min (Table 1).

**Table 1:** Demographic characteristics of participants

Variables	Frequency	Percent
Gender		
Female	10	16.7
Male	50	83.3
Ward		
ICU	55	91.7
CCU	5	8.3
Systolic blood pressure (mmHg)		
< 120 mmHg	20	35
120 mmHg	10	16.7
> 120 mmHg	29	48.3
diastolic blood pressure (mmHg)		
< 80 mmHg	27	45
80 mmHg	7	11.7
> 80 mmHg	26	43.3
Pulse rate (beats per minute)		
> 60	8	13.3
60–80	14	23.3
> 80	38	63.3
Saturation of O_2_ (mmHg)		
< 90	9	15
90–96	27	45
> 96	24	40
GCS score		
3–5	20	33.3
5–7	40	66.7
Age (years)	Mean	SD
	47.06	19.11

The results revealed that the sensitivity, specificity and accuracy of the capnography were higher than those of the epigastric auscultation. Capnographic sensitivity of 100%, specificity of 92.5%, and positive and negative predictive values of 86.9 and 100% were reported at 95% accuracy (Table 2).

**Table 2:** sensitivity, specificity, PPV, NPV and accuracy of epigastric auscultation and capnography in assessing the nasogastric tube placement in the ICU patients

Variables	Sensitivity%	Specificity%	PPV%	NPV%	Accuracy%
Capnography	100	92.5	86.9	100	95
Epigastric auscultation	90	80	69.2	94.1	83.4

The Kappa agreement coefficient was used to determine the agreement between two methods in the correct placement of the gastric tube. In terms of agreement between variables of capnography)respiratory placement of nasogastric tube(and epigastric auscultation)respiratory placement of nasogastric tube(, the percentage of agreement was 80.8%. Also, for variables of capnography) gastric placement of nasogastric tube(and epigastric auscultation) gastric placement of nasogastric tube (, the agreement was 94.1%; according to Kappa agreement, significant results were obtained (*p* < 0.05) (Table 3).

**Table 3:** Kappa agreement coefficient of epigastric auscultation and capnography in assessing the nasogastric tube placement in the ICU patients

Variables	Capnography		Statistic index
	respiratory placement of nasogastric tube	Gastric placement of nasogastric tube	
	Frequency (%)	Frequency (%)	
Epigastric auscultation			
respiratory placement of nasogastric tube	21 (80.8)	5 (19.2)	Fisher’s = 34.95 Exact Test P-value = 0.001 −0.759 Measure of Agreement (Kappa) *P*-value = 0.001
Gastric placement of nasogastric tube	2 (5.9)	32 (94.1)	

**Fig. 1:**
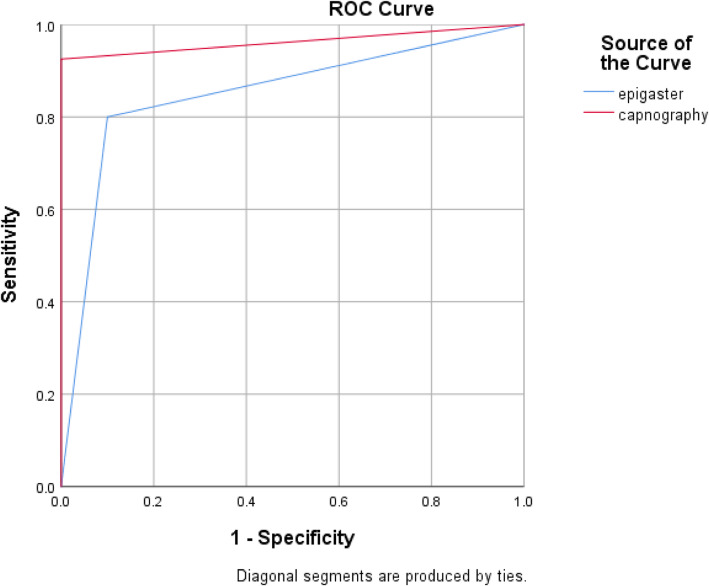
Area under ROC curve of capnography and epigastric auscultation

The area under ROC curve of capnography and epigastric auscultation showed a better performance of capnography (Fig. 1).

## Discussion

According to the results of the current study, capnography had a higher sensitivity, specificity, and accuracy, compared to the epigastric auscultation technique. In a research, Kindopp et al. evaluated capnography in determining feeding tube insertion in ICU patients. According to the results, the use of capnography for determining the location of the NG tube inserted in the airway had 100% sensitivity and specificity. Thus, capnography can be employed in placing the NG tube to increase patient safety [25]. In another study, Haghighi Moghadam et al. [26]. noted that capnography had 100% accuracy in distinguishing the airway from the gastrointestinal tract, where capnography results had 100% correlation with radiography results, the golden standard for determining the location of the NG tube. As such, capnography can be used as a substitute for radiography to distinguish the gastrointestinal tract from the airway during NG tube insertion [1]. In another study by Bruns et al., capnography was applied to determine the location of the NG tube as a simple, low-cost, and reliable method [5].

While capnography results are appropriate in studies, they are presented as false positive when the NG tube is left in the mouth or the throat. Some studies also confirm this claim [12, 25], and if the tube does not enter the stomach sufficiently and remains in the esophagus, the capnography will show the results as false positive, in which case the risk of aspiration will increase [5]. Our findings are somehow different from similar studies. In a study by Rahimi et al., the diagnostic percentage of epigastric auscultation was 33.3% in the detection of tube insertion in the lung and 29.2% in gastric tube insertion. These scholars expressed that despite the widespread use of epigastric auscultation, it is not a reliable technique [6]. In spite of a low detection of feeding tube insertion with epigastric auscultation, this method is used relatively frequently. In a research by Roynette et al., which was performed in 383 ICUs of 20 European countries, the results demonstrated that 84.7% of nurses applied the epigastric auscultation method [27]. In another study, the results indicated that 86% of nurses employed the epigastric auscultation technique to confirm the feeding tube insertion [28].

Some studies have evaluated both epigastric auscultation and capnography in confirmation of the feeding tube insertion. In a research by Meyer et al., the accuracy of a combined method of epigastric auscultation and colorimetric capnography was evaluated in confirming the correct location of the NG tube in ICU patients connected to a mechanical ventilator. Carbon dioxide was observed in 9 out of 69 cases of NG tube insertion, two and seven of which were placed in the airway and the stomach, respectively. Nevertheless, the findings indicated a complete correlation between colorimetric capnography and epigastric auscultation for confirming the location of the NG tube. Further, the present research revealed that colorimetric capnography along with epigastric auscultation is a safe and low-cost method that promotes health care for patients [29]. In a study by Galbois et al., the results indicated that the use of capnography and epigastric auscultation reduced the costs and time required for determining the location of the NG tube, and that these are safe and cost-effective techniques for improving nursing care [15].

In a previous research, Erzincanli et al. evaluated colorimetric capnometry in confirming the NG tube insertion, reporting a 95% correlation between colorimetric capnography and radiography and an 82.5% correlation between epigastric auscultation and radiography. Furthermore, the results of the mentioned study revealed 100% sensitivity and 66.7% specificity of colorimetric capnography in confirming the insertion of the NG tube. While colorimetric capnography has been successful in the distinguishing of the airway from the respiratory tract, it fails to separate the tube inserted in the stomach and/or duodenum [22]. Alpiren et al. conducted a research to compare the accuracy of capnometry and epigastric auscultation in the placement of NG tube to determine the correct place of NG tube in ICU patients. These scholars reported that capnometry detected wrong location in 15 (16%) out of 91 cases of tube insertion (lung instead of stomach), while epigastric auscultation detected the wrong location in five (5%) out of 91 cases of tube insertion (lung instead of stomach), which is inconsistent with our findings. The results obtained demonstrated that neither epigastric auscultation nor capnometry were reliable methods for confirming the NG tube insertion, and chest radiography was the only reliable technique for determining the accurate location of the tube [1]. This lack of consistency between the results might be due to neglecting the time for removal or absorption of carbon dioxide present in the stomach. In the current research, 1 min was considered for absorption or removal of carbon dioxide in the stomach through the NG tube.

### Limitations

The limitations of this study were the low sample size and low level of consciousness of patients, which can reduce the generalizability of findings.

## Conclusion

According to the results, both techniques are simple and inexpensive, but the use of capnography is more preferred over epigastric auscultation. However, none of these methods can absolutely predict the correct insertion of the NG tube. Thus, it is recommended that more than one method be applied to detect and confirm the insertion of the NG tube and a combination of both capnography and epigastric auscultation.

## Data Availability

Datasets are available through the corresponding author upon reasonable request.
